# Discovery of biomarkers for glycaemic deterioration before and after the onset of type 2 diabetes: rationale and design of the epidemiological studies within the IMI DIRECT Consortium

**DOI:** 10.1007/s00125-014-3216-x

**Published:** 2014-04-04

**Authors:** Robert W. Koivula, Alison Heggie, Anna Barnett, Henna Cederberg, Tue H. Hansen, Anitra D. Koopman, Martin Ridderstråle, Femke Rutters, Henrik Vestergaard, Ramneek Gupta, Sanna Herrgård, Martijn W. Heymans, Mandy H. Perry, Simone Rauh, Maritta Siloaho, Harriet J. A. Teare, Barbara Thorand, Jimmy Bell, Søren Brunak, Gary Frost, Bernd Jablonka, Andrea Mari, Tim J. McDonald, Jacqueline M. Dekker, Torben Hansen, Andrew Hattersley, Markku Laakso, Oluf Pedersen, Veikko Koivisto, Hartmut Ruetten, Mark Walker, Ewan Pearson, Paul W. Franks, for the DIRECT Consortium

**Affiliations:** 1 Department of Clinical Sciences, Lund University, Genetic and Molecular Epidemiology, CRC, Skåne University Hospital Malmö, Building 91, Level 10, Jan Waldenströms gata 35, SE-205 02 Malmö, Sweden; 2 Institute of Cellular Medicine (Diabetes), The Medical School, Newcastle University, Framlington Place, Newcastle upon Tyne, NE2 4HH UK; 3 Division of Cardiovascular & Diabetes Medicine, Medical Research Institute, University of Dundee, Dundee, DD1 9SY UK; 4 Department of Medicine, University of Eastern Finland and Kuopio University Hospital, Kuopio, Finland; 5 The Novo Nordisk Foundation Center for Basic Metabolic Research, Section of Metabolic Genetics, Faculty of Health Sciences, University of Copenhagen, Copenhagen, Denmark; 6 Department of Epidemiology and Biostatistics, VUmc, Amsterdam, the Netherlands; 7 EMGO+ Institute for Health and Care Research, VUmc, Amsterdam, the Netherlands; 8 Department of Clinical Sciences, Clinical Obesity, Skåne University Hospital Malmö, Malmö, Sweden; 9 Steno Diabetes Center, Gentofte, Denmark; 10 Center for Biological Sequence Analysis, Department of Systems Biology, Technical University of Denmark, Kongens Lyngby, Denmark; 11 NIHR Exeter Clinical Research Facility, University of Exeter, Exeter, UK; 12 Blood Sciences, Royal Devon and Exeter NHS Foundation Trust, Exeter, UK; 13 HeLEX, Nuffield Department of Population Health, University of Oxford, Oxford, UK; 14 Institute of Epidemiology II, Helmholtz Zentrum Muenchen, German Research Center for Environmental Health (GmbH), Neuherberg, Germany; 15 German Center for Diabetes Research (DZD), Neuherberg, Germany; 16 Metabolic and Molecular Imaging Group, MRC Clinical Science Centre, Imperial College Hammersmith Campus, London, UK; 17 Division of Endocrinology and Metabolism, Nutrition and Dietetic Research Group, Imperial College London, London, UK; 18 Sanofi-Aventis Deutschland GmbH, R&D, Frankfurt am Main, Germany; 19 Institute of Biomedical Engineering, National Research Council, Padova, Italy; 20 Faculty of Health Sciences, University of Southern Denmark, Odense, Denmark; 21 Genetics of Complex Traits, University of Exeter Medical School, Exeter, UK; 22 Genetics of Diabetes, University of Exeter Medical School, Exeter, UK; 23 Hagedorn Research Institute, Gentofte, Denmark; 24 Institute of Biomedical Science, Faculty of Health Sciences, University of Copenhagen, Copenhagen, Denmark; 25 Eli Lilly & Company, Helsinki, Finland; 26 Department of Nutrition, Harvard School of Public Health, Boston, MA USA; 27 Department of Public Health & Clinical Medicine, Section for Medicine, Umeå University Hospital, Umeå, Sweden

**Keywords:** Epigenetic, Gene–environment interaction, Genome, Glycaemic control, Lifestyle, Microbiome, Prediabetes, Proteome, Transcriptome, Type 2 diabetes

## Abstract

**Aims/hypothesis:**

The DIRECT (Diabetes Research on Patient Stratification) Study is part of a European Union Framework 7 Innovative Medicines Initiative project, a joint undertaking between four industry and 21 academic partners throughout Europe. The Consortium aims to discover and validate biomarkers that: (1) predict the rate of glycaemic deterioration before and after type 2 diabetes onset; (2) predict the response to diabetes therapies; and (3) help stratify type 2 diabetes into clearly definable disease subclasses that can be treated more effectively than without stratification. This paper describes two new prospective cohort studies conducted as part of DIRECT.

**Methods:**

Prediabetic participants (target sample size 2,200–2,700) and patients with newly diagnosed type 2 diabetes (target sample size ~1,000) are undergoing detailed metabolic phenotyping at baseline and 18 months and 36 months later. Abdominal, pancreatic and liver fat is assessed using MRI. Insulin secretion and action are assessed using frequently sampled OGTTs in non-diabetic participants, and frequently sampled mixed-meal tolerance tests in patients with type 2 diabetes. Biosamples include venous blood, faeces, urine and nail clippings, which, among other biochemical analyses, will be characterised at genetic, transcriptomic, metabolomic, proteomic and metagenomic levels. Lifestyle is assessed using high-resolution triaxial accelerometry, 24 h diet record, and food habit questionnaires.

**Conclusions/interpretation:**

DIRECT will yield an unprecedented array of biomaterials and data. This resource, available through managed access to scientists within and outside the Consortium, will facilitate the development of new treatments and therapeutic strategies for the prevention and management of type 2 diabetes.

**Electronic supplementary material:**

The online version of this article (doi:10.1007/s00125-014-3216-x) contains peer-reviewed but unedited supplementary material, which is available to authorised users.

## Introduction

Type 2 diabetes is a highly prevalent disease that is usually preceded by four pathophysiological phases: (1) a gradually accumulating resistance to the peripheral effects of insulin on cellular glucose transportation; (2) a compensatory rise in endogenous insulin secretion; (3) a progressive failure of beta cell function; (4) a corresponding loss of glycaemic control that eventually manifests as type 2 diabetes. The order of these phases can differ, with the primordial defect in certain high-risk populations appearing to be at the level of the pancreatic beta cell rather than in peripheral cells [1]. In patients with type 2 diabetes, clinical interventions initially focus on regaining glucose homeostasis through lifestyle-induced weight loss and/or with pharmacotherapies designed to reduce hepatic glucose production (e.g. metformin), enhance beta cell function (e.g. sulfonylureas), reduce glycogen concentrations (e.g. gliptins) or sensitise peripheral cells to the effects of insulin (e.g. thiazolidinediones). Each therapy is designed to directly or indirectly enhance the function of the remaining beta cells and/or improve insulin action; however, beta cell mass and function usually continue to decline after the therapy is initiated and thereafter the therapy’s effectiveness lessens as the time since diagnosis increases. Indeed, roughly 25% of patients with type 2 diabetes require exogenous insulin within 6 years of diagnosis, and 42% within 10 years [2].

Although the diagnosis of type 2 diabetes is straightforward, determined primarily on the basis of elevated blood glucose concentrations, it is a diagnosis of exclusion, such that it is diagnosed when no other plausible cause is known. Patients vary greatly in their clinical characteristics, treatment requirements, rate of glycaemic deterioration, and susceptibility to diabetic complications. As we discover more about the aetiology of diabetes, it is likely that this group with ‘type 2 diabetes’ will be reclassified into other subclasses of diabetes with different pathophysiologies. If these pathophysiological subclasses could be identified before or around the time of diagnosis, this information might help facilitate targeted interventions, which in turn might lead to improved treatment effectiveness, a reduction in unnecessary side effects, less costly treatments, better patient adherence to treatments, and improved quality of life. The identification of biomarkers that aid therapeutic targeting in prediabetes (Study 1) or early-onset type 2 diabetes (Study 2) is a major innovative objective of the DIRECT (Diabetes Research on Patient Stratification) Consortium. The term ‘prediabetic’ is used in our paper because the target population of Study 1 was identified using a risk prediction algorithm that explicitly seeks to identify men and women who are in the prediabetic blood glucose or HbA_1c_ ranges and who are at elevated risk of rapid glycaemic deterioration (see Methods). Therefore, whilst many persons within the general population who are defined to have ‘prediabetic’ levels of blood glucose will regress to normal glycaemia, most in the Study 1 population are, by selection, likely to go on to develop diabetes. We use the term ‘prediabetes’ with this assumption from hereon.

The DIRECT Consortium was formed under the banner of the Innovative Medicines Initiative (IMI), a joint undertaking between the European Union (EU), European academic institutions and pharmaceutical companies that forms part of the Seventh Framework Programme (FP7). The overarching objectives of the DIRECT Consortium are to identify biomarkers that address current bottlenecks in diabetes drug development and to develop a stratified medicines approach to treatment of type 2 diabetes with either existing or novel therapies. The DIRECT Consortium seeks to address two key areas in which improvements in diabetic medicine are required: (1) the rate at which peoples’ glycaemic control deteriorates either from prediabetes to type 2 diabetes or through type 2 diabetes with increasing treatment requirements; (2) the extent to which certain therapeutic interventions (either pharmacological or surgical) result in improved glycaemic control.

**Fig. 1:**
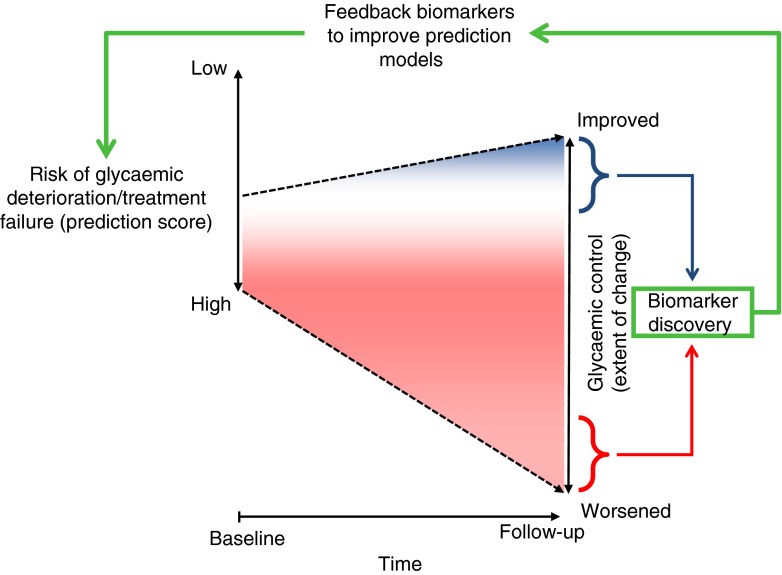
Overview of the biomarker discovery strategy in the two prospective studies of the DIRECT consortium (Studies 1 and 2). Persons at high risk of glycaemic deterioration before (Study 1) or soon after (Study 2) the onset of type 2 diabetes are enrolled and followed for between 18 and 36 months. Those whose glycaemic control deteriorates least and most are selected for biomarker discovery. Discovered biomarkers are subsequently fed back to improve risk prediction models, which will be validated in other epidemiological studies and clinical trials organised by the DIRECT Consortium and its partners

This paper concerns two multicentre prospective cohort studies within DIRECT, which address the area of glycaemic deterioration. These studies focus on amassing information and biomaterials that will be used to discover novel biomarkers for glycaemic deterioration in people at high risk of developing type 2 diabetes (Study 1) and in those who have recently been diagnosed with the disease (Study 2) (see Fig. 1 and http://www.direct-diabetes.org/). This paper overviews the design and rationale of these two studies, in addition to a description of approaches for biomarker discovery focused on existing cohort studies that are accessible to the Consortium.

## Methods

**Fig. 2:**
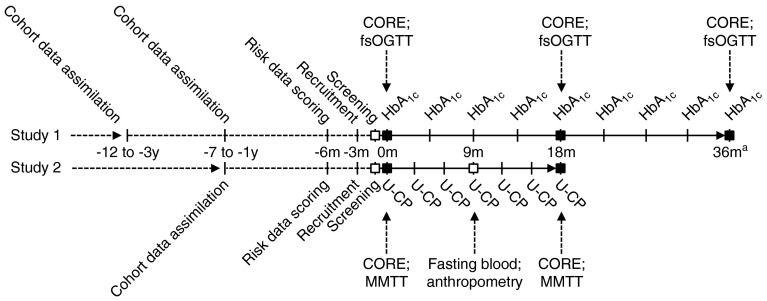
Overview of the timeline of the DIRECT WP2 Study 1 and Study 2 protocols. Core assessments (CORE) are: anthropometry; fasting blood; MRI^b^; faecal microbiome; urine; physical activity; diet; quality of life; diabetes family history; medication history. Dashed lines indicate data assimilated from existing cohorts and registers. m, months; U-CP, urinary C-peptide; y, years. ^a^Only a subset of the original sample population will be invited to attend the 36m visit; ^b^carried out in a subset of the sample population. Black squares, deep-phenotype study visit; white squares, minor study visit

The glycaemic deterioration work package (WP2) of the DIRECT Study comprises four sub-studies, two of which involve the collection of new data. Study 1 concerns recruitment and follow-up of about 2,200–2,700 people with prediabetes to study glycaemic deterioration before diabetes diagnosis. Deep phenotyping will be performed at baseline and after 18 and 36 months. Study 2 will address glycaemic deterioration in about 1,000 patients with new-onset type 2 diabetes. Deep phenotyping will be performed at baseline and after 18 months. Additional work in WP2 of the DIRECT Study, which will not be discussed in depth here, will make use of data from pre-existing cohorts [3–12]. The protocol timeline for the visits and tests for Study 1 and Study 2 are shown in Fig. 2.

### Ethical, regulatory and legal considerations

As the DIRECT Study clinical centres are located across Europe, in the absence of a pan-European unifying body for research ethics approval, partners in each of the countries represented in the DIRECT Consortium navigated their local research ethics processes separately. This was necessary to ensure that the DIRECT protocols conformed with each DIRECT centre’s ethical, regulatory and legal requirements. As well as adhering to external EU, national and local governance structures, an internal governance structure was implemented to support the participant consent form sections relating to privacy, data sharing and data security, which were established in consultation with all DIRECT partners. A committee was established to develop policies relating to the handling of samples and data. To ensure that participant privacy and data security standards are maintained, while not unnecessarily impeding the research process and ensuring equity for all research partners, a Data Access Committee made up of Consortium members was formed, which is responsible for reviewing, approving and enabling access to data by research partners.

### Methods common to Study 1 and Study 2

#### Anthropometric and blood pressure

All measurement procedures are standardised across study sites and performed by trained nurses or research assistants. Height is measured using calibrated wall-mounted stadiometers, weight using calibrated scales, and waist, hip, thigh and calf circumferences using non-stretchable measuring tapes. Blood pressure is measured using calibrated manual (or automatic) sphygmomanometers with an appropriately sized arm cuff; three seated measures are recorded in each participant. Some centres also estimate body composition using bioimpedance scales, although this is not a requirement of the core protocol.

#### Blood omics

Fasting blood samples are taken in both studies for genomic, epigenomic, transcriptomic, proteomic and metabolomic assessments. These analyses are carried out to allow systems-based investigations, which will be a major feature of the DIRECT Study [13]. A variety of omics data will be generated with validated methods to be defined in subsequent studies depending on the hypotheses being tested. The omics methods used will hence be described in detail in subsequent papers.

#### Beta cell function and insulin sensitivity

Beta cell function and insulin sensitivity will be assessed using validated modelling methods based on an OGTT or a mixed-meal tolerance test (MMTT), as well as empirical indices [14, 15].

#### Microbiome assessment

A faecal sample for DNA isolation and metagenome deep sequencing is collected under standardised conditions. These samples are immediately frozen at home and transported to the clinical research centres in cooled packages. The samples are subsequently stored at −80°C until DNA extraction. Bacterial DNA will be extracted following standardised consensus procedures and subjected to deep metagenomic next generation sequencing, as previously described [16]. For each sample, 3 Gb clean sequence data are generated, and, on the basis of bacterial gene annotation, principal component and cluster analyses are performed. Taxonomic classification of known species, metagenomic assessment of unknown species, and functional-potential analyses are undertaken to identify changes in the gut microbiota composition and function and to correlate such signatures with a series of biochemical and physiological variables of the host [16].

#### Urine sample

A fasting urine sample is taken and a pregnancy test is performed in premenopausal female participants. A dipstick test is also performed on all urine samples using Multistix 10 (Bayer, Leverkusen, Germany). Samples are aliquoted into plain tubes and stored at −80°C.

#### MRI

Magnetic resonance images are acquired on approximately every second participant in sequence, within age (5 years) and sex (male and female) strata, to ensure an approximately even distribution of scans of men and women and across the spectrum of age in both studies at five locations throughout northern Europe. Local protocols were standardised across study centres by an experienced radiographer to harmonise the scan methodology as far as possible given that each centre has different equipment. Scans are made at 1.5 and 3.0T field strengths, depending on equipment, and using different manufacturers’ scanner models: Siemens Trio 3T (University of Dundee, UK), Philips Intera 1.5T (University of Exeter, UK), Siemens Espree 1.5T (University of Newcastle, UK), Philips Achieva 3T (Copenhagen University, Denmark) and Siemens Avanto 1.5T (University of Eastern Finland, Finland and VU University Medical Center, Amsterdam, the Netherlands).

##### Abdominal MRI

All participants are scanned in the prone position with arms extended above the head. T1-weighted images are acquired from the diaphragm to acetabulum using the maximum field of view during free breathing (slice thickness of 10 mm, with a slice gap of 10 mm).

##### Pancreatic volume

Imaging of the pancreas is achieved following additional survey scans while in suspended respiration. A three-dimensional T1-weighted scan with fat suppression is placed over the pancreas to cover the entire organ. A block of 50–80 slices with a thickness ranging from 1.2 to 2 mm is used depending on scanner limitations. Scans are performed in a single breath-hold on expiration.

##### Multi-echo for pancreatic liver fat

The pancreas is identified and further axial images are performed during suspended respiration, which are used to position a single slice multi-echo sequence through the pancreas using a surface coil. Typical variables include: repetition time, 1,500 ms; field of view, 500; slice thickness, 10 mm. Echo times vary between 8 and 20 ms, depending on the scanner used, and are chosen to represent in and out of phase, the shortest acquired at 1.15 ms and the longest at 23 ms. An identical single slice is then acquired through the liver in the axial plane. This method has been appropriately validated previously [17].

Raw data are converted into an analysable format using Image J (Image; National Institutes of Health, Bethesda, MD). An automated pixel-by-pixel analysis is performed to obtain colour-coded parametric maps of the entire pancreas and liver using Matlab version 7.7 (Mathworks, Natick, MA, USA). Relative proportions of fat and water within each organ are then calculated.

#### Dietary assessment

For the assessment of diet and nutrition, a 24 h multi-pass dietary record is used. Diet assessments in each participant are made the day before the study visit. Twenty-four hour multi-pass dietary records are open-ended, do not require a high degree of literacy, and impose a relatively low level of burden on the respondent. The method is well validated against the gold standard for energy intake quantification (double-labelled water) and has good reproducibility [18–20]. The methods for the dietary record and the food habit questionnaire have been validated as part of the Euroaction Study [21]. The method is structured into three levels of dietary questioning or ‘passes’. The first pass aims to document a ‘usual’ day’s meal. The second pass aims to give the respondent the time to reflect and add to the foods recorded in the first pass. The third pass of the food record aims to obtain information about portion size and method of preparation using a food portion size atlas. Participants also complete a food habit questionnaire to assess the overall quality of the diet against healthy eating and diabetes guidelines, and as an internal quality control check for the record. Toenail clippings for the objective assessment of trace elements are also collected using stainless-steel clippers and are stored in paper envelopes in a cool, dry environment.

Analysis of diet data will be undertaken using Dietplan-6, a comprehensive food analysis program (version 6.70.43, 2013; Forestfield Software, Horsham, UK). Because Studies 1 and 2 are pan-European, diet questionnaires were written in several languages. Thus, each questionnaire is translated into English by a native speaker of the respective non-English language. Micro- and macro-nutrient content are calculated in a way that preserves the meal structure. Each individual’s questionnaire contains the contribution of each food’s nutritional content related to the total intake in the meal structure. Under- and over-reporting of energy intake will be assessed using Goldberg’s equation [22, 23].

#### Physical activity, sedentary behaviour and sleep assessment

Habitual physical activity is assessed using a wrist-worn triaxial accelerometer (ActiGraph GT3X+; Actigraph LLC, Pensacola, FL, USA). The monitor is fitted to the participant’s non-dominant wrist using an adjustable strap (Actigraph LLC). The participant is requested to wear the monitor continuously for 10 days to allow habitual uninterrupted measures of both sleep and physical activity. The monitor is set to record at 30 Hz with the manufacturer’s sleep mode disabled. Participants selected for detailed assessments at the 18-month and final 36-month study visit (in Study 1) will wear an additional monitor on their dominant hip. The participants are instructed to remove the monitor only when undertaking water-based activities (deeper than 1 m and lasting longer than 30 min), or if the monitor causes discomfort. Participants are given a prepaid, addressed, padded envelope in which to deposit the monitor and return it. A wide variety of methods can be used to analyse the raw data to provide meaningful summary variables. The analytical methods used will depend on the specific research question and will be described in detail in subsequent papers.

#### Additional questionnaires

Questionnaire data are also collected on quality of life (SF12), dental health status, family history of diabetes and medication history.

#### Central database

A relational database has been constructed to store all foreground data collected in the DIRECT project. This includes a user-friendly interface for creating participant profiles, data entry from case report forms, importing processed laboratory and measurement data, and for data query. All samples are barcoded, linked to the participant’s study identification number, and registered in the database. A comprehensive sample and data-tracking approach ensures that samples can be localised to the specific placeholder in a rack at a specified storage facility, which is an important infrastructural feature of the project that will minimise data loss due to human error. Additional functions for data interrogation, reporting errors and anomalies, and repair processes are built into the database. The database was constructed using mySQL (version 5.1.46). The backend was built using Perl (version 5.12.1), and the frontend was built using Javascript (version 1.8.5) and jQuery (version 1.7.1).

#### Statistical power

There are many possible scenarios under which analyses will be performed in DIRECT. The following is merely an example to illustrate the statistical power for one such scenario for prediabetic participants. Analysis will involve linear regression analysis where we model the ability of a given biomarker to predict change in a continuous trait outcome. Power will vary depending on a variety of factors. However, we can calculate an example where we use a range of biomarker frequencies to show power for detecting difference in change in glucose concentrations (population mean ± SD, 5.8 ± 0.6 mmol/l) during the study. Where a dichotomous biomarker is present in 10% of the cohort, we will have 99.9%, 99.9% and 73.9% power (α 0.05) to detect changes in fasting glucose of 0.5 mmol/l, 0.2 mmol/l and 0.1 mmol/l, respectively. If the biomarker is more common and the other assumptions outlined above remain the same, power exceeds 95% in all examples. The power calculations above assume a single hypothesis test. In DIRECT, we are likely to undertake many thousands of hypothesis tests; therefore, determining true from false positive findings will rely on replication studies (in existing epidemiological cohorts linked to DIRECT) and validation studies (in clinical trials that will be undertaken in the second stage of the DIRECT project).

### Prediabetic glycaemic deterioration (Study 1)

#### Study 1 rationale

The primary objective of Study 1 is to collect biosamples and information that might yield novel, predictive biomarkers for glycaemic deterioration in non-diabetic high-risk participants.

#### Study 1 participant identification

Participants in Study 1 were recruited from existing prospective cohort studies in or around each of the following European cities: Malmö, Sweden (Malmö Diet and Cancer Study [12]); Amsterdam, The Netherlands (Hoorn Study [3]); Copenhagen, Denmark (Inter99 [10]); and Kuopio, Finland (METSIM [24]). A clinically practicable screening tool (DIRECT-DETECT) was used to identify at-risk participants from existing cohort studies, who were then recruited into this new prospective cohort study (Study 1). Inclusion and exclusion criteria for Study 1 are outlined in Table 1.

**Table 1:** Inclusion and exclusion criteria for Study 1

Inclusion criteria
• No treatment with insulin-sensitising, glucose-lowering or other antidiabetic drugs
• Fasting capillary blood glucose <10 mmol/l at baseline
• White European (self-report of parental ethnicity)
• Age ≥35 and <75 years
Exclusion criteria
• Diagnosed diabetes of any type, HbA_1c_ ≥6.5% (48 mmol/mol) or fasting plasma glucose ≥7.0 mmol/l or 2 h plasma glucose >11.0 mmol/l previously
• For women, pregnancy, lactation or plans to conceive within the study period
• Use of a pacemaker
• Any other significant medical reason for exclusion as determined by the investigator

Participants invited to attend the baseline visit are defined as ‘prediabetic’ based on HbA_1c_ (5.7–6.4%, 40–48 mmol/mol). Participants with HbA_1c_ values at baseline ≥6.5%, or who are known to have prevalent diabetes according to the ADA 2011 criteria [25], are excluded. Participants who are clinically diagnosed with diabetes during the course of the study will be allowed to remain in the study provided that their fasting blood glucose levels do not exceed 10 mmol/l at a follow-up visit. Treatments and medications for diabetes and other indications are recorded. Those participants who have started antidiabetic medications are asked to stop taking them 24 h before the 18- and 36-month follow-up examinations to minimise their effects on the interpretation of drug-sensitive biomarkers.

Like other diabetes screening tools, DIRECT-DETECT is neither 100% specific nor sensitive; thus, we anticipate that, although on average glycaemic control will decline in the cohort, the extent to which this occurs will vary widely across the cohort. Thus, participants in the top and bottom quantiles (*n* = 300 per quantile) of the distribution of glycaemic change in Study 1 will be selected for deeper phenotyping, and comparisons will be made to identify biomarkers that differ substantially between the two groups, and hence might improve the predictive accuracy of the DIRECT-DETECT risk prediction algorithms (see Fig. 1).

##### Development of the DIRECT-DETECT prediction model

The prediction tool comprises two models. The focus of the first model is to identify non-diabetic individuals who are at high risk of rapid, short-term glycaemic deterioration, predicted by questionnaire variables only. The focus of the second model is to further predict glycaemic deterioration by adding a recent HbA_1c_ measure to the model. The DIRECT-DETECT tool is an adapted version of the DETECT-2 algorithm [26]. To create the tool, data from three existing prospective cohort studies (Hoorn Study, Cooperative Health Research in the Region of Augsburg [KORA S4/F4 Study] and Inter99 Study; cohort characteristics are presented in Table 2 and published in detail elsewhere [3, 4, 10]) were used to model the relationships between selected variables determined at baseline (age, BMI, waist circumference, use of antihypertensive medication, smoking and parental diabetes) and change in HbA_1c_ during the following 4–8 years.

**Table 2:** Description of the characteristics of the studies used to develop the DIRECT-DETECT prediction model

Characteristic	All studies (*n* = 6,136)	Hoorn (*n* = 1,345)	KORA S4/F4 (*n* = 904)	Inter99 (*n* = 3,887)
Age (years)	52 (30–79)	60 (50–77)	64 (55–74)	46 (30–61)
Sex (% male)	49%	46%	51%	50%
HbA_1c_ at follow-up (%)	5.66 ± 0.44	5.47 ± 0.62	5.64 ± 0.41	5.73 ± 0.34
HbA_1c_ at follow-up (mmol/mol)	38.4 ± 4.8	36.3 ± 6.8	38.1 ± 4.5	3.91 ± 3.7
Follow-up duration (years)	6 (4–8)	6.5 (4–8)	7 (6.5–8)	5.5 (5–6.5)

Values are mean (range) or mean ± SD

The prediction models were developed in men and women separately using linear regression equations. The models were evaluated for their calibrative and discriminative abilities using calibration plots (to assess agreement between the predicted and observed values of HbA_1c_) and by selecting participants with the highest 50% observed and predicted HbA_1c_ values within whom sensitivity, specificity, and positive and negative predictive values were assessed respectively. The prediction models were internally validated using bootstrapping techniques. External validation of the model was performed in the METSIM cohort.

#### Study 1 screening examination

Using the DIRECT-DETECT tool, a total of around 10,000 high-risk participants have been identified from the existing population-based prospective cohort studies at each study centre. The participants are ranked according to the DIRECT-DETECT model scores, and the highest-ranking are invited to attend a screening examination. A detailed description of the risk prediction model will be published elsewhere. To ensure that only high-risk individuals are invited to the full study, updated information on the prediction variables and information on inclusion/exclusion criteria are obtained, and HbA_1c_ is measured at the screening examination. In some centres, fasting and/or 2 h glucose concentrations are also measured at this stage and used for the same purpose as HbA_1c_. Participants who satisfy the inclusion/exclusion criteria (see Table 1) and have been identified as being at high risk of glycaemic deterioration from retrospective cohort data are invited to attend the baseline visit. Participants with apparently normal glycaemia who are determined to be at high risk on the basis of the DIRECT-DETECT second model score are also invited to participate in the full study. The intention is to enrol between 2,200 and 2,700 of the highest-risk persons across all DIRECT Study centres.

#### Study 1 baseline examination

Examinations are carried out the morning after a 10 h overnight fast. Study-specific written, informed consent is obtained in person. Anthropometrics and blood pressure are measured. A stool sample, toenail clippings and urine sample are collected. The participant is fitted with an accelerometer (as outlined above) for measurement of physical activity, sedentary behaviour and sleep. Data on quality of life (SF12) and diet (as outlined above) are obtained by questionnaire. Abdominal MRI scans (as outlined above) are conducted. All current medication including over-the-counter and herbal medication is documented.

##### Frequently sampled OGTT (fsOGTT)

For the collection of blood, a cannula is inserted into a forearm vein for sampling at time points 0, 15, 30, 45, 60, 90 and 120 min during the 75 g fsOGTT. Blood samples for downstream blood omics processing (as described above) are extracted and stored. A standard finger-stick fasting capillary blood glucose sample (with a HemoCue Glucose 201 or similar) is taken before the fsOGTT, and those with corrected fasting venous glucose >10 mmol/l (capillary glucose >11 mmol/l) are excluded from the study. To adjust for difference between plasma glucose and capillary blood glucose, a correction factor of ~1.11 is applied manually or automatically, as per most commercially available capillary blood glucose meters [27].

#### Study 1 measurements between core examinations

All participants are provided with a finger-stick blood sampling kit for the collection of a blood spot (as described above) for HbA_1c_ assessment, a non-stretchable tape measure for the assessment of waist circumference, and a data collection form for recording waist circumference and body weight. Participants are asked to use the same weighing scales throughout the study so that home measurements can be calibrated with clinical measurements. Each measurement is obtained at 18-week intervals from the baseline visit to the follow-up visit 18 months later (a total of three intermediate measurements). The participant is requested to return the blood spots and questionnaires to the study centre by surface mail in a prepaid envelope. Blood spot measures are processed according to a validated protocol [28].

#### Study 1 follow-up examinations at 18 and 36 months

Similar to the baseline visit, anthropometric, diet and quality-of-life data, blood pressure, stool samples, toenail clippings and urine samples are collected. The participant is fitted again with the accelerometer for measurement of physical activity, sedentary behaviour and sleep. Fasting and fsOGTT blood samples are obtained. At the 18-month follow-up visit, the 300 participants at each end of the glycaemic change distribution (*n* = 600) are selected from the full cohort (*N* = 2,200–2,700) for additional deep phenotyping. The cut-off points for the tails in the rate of glycaemic deterioration are determined using data from the sequential HbA_1c_ data collected during the 18-month follow-up period. Because participants within the tails of this distribution will be identified as study data is accrued, and many of these will be identified before all of the sequential HbA_1c_ data are available, it will be necessary to predict the distribution of glycaemic change using all of the available data at that time. This dataset will be continuously updated with additional HbA_1c_ data, thereby maximising the correct classification of participants included in the deep-phenotyped subgroup of Study 1. These participants have a further MRI scan. An additional hip-worn accelerometer is also fitted to these individuals to further assess physical activity, sedentary behaviour and sleep.

### New-onset diabetes glycaemic deterioration (Study 2)

#### Study 2 rationale

The primary objective of Study 2 is to collect biosamples and information that might yield novel, predictive biomarkers for glycaemic deterioration in people who have recently been diagnosed with type 2 diabetes.

#### Study 2 participant identification

Participants in Study 2 of DIRECT are recruited from or nearby each of the following European cities: Malmö, Sweden; Amsterdam, the Netherlands; Copenhagen, Denmark; Exeter, UK; Newcastle, UK; Dundee, UK. Potential participants are recruited through targeted searches of existing databases and research registers combined with person-to-person contact at educational clinics and through routine retinal screening programmes. The target sample size is 1,000 participants evenly distributed across the six European centres. Inclusion and exclusion criteria for Study 2 are shown in Table 3.

**Table 3:** Study 2 inclusion and exclusion criteria

Inclusion criteria
• Patients diagnosed with type 2 diabetes not less than 6 months and not more than 24 months before baseline examination
• Management by lifestyle with or without metformin therapy
• All HbA_1c_ <7.6% (<60 mmol/mol) within previous 3 months
• White European
• Age ≥35 and <75
• Estimated GFR >50 ml/min
Exclusion criteria
• Type 1 diabetes
• A previous HbA_1c_ >9.0% (>75 mmol/mol)
• Prior treatment with insulin or an oral hypoglycaemic agent other than metformin
• BMI <20 or >50 kg/m^2^
• Pregnancy, lactation or plans to conceive within the study period
• Any other significant medical reason for exclusion as determined by the investigator

#### Study 2 screening examination

Participant eligibility is assessed, and written informed consent is obtained in person. Additional information on diabetic complications and other comorbidities, family history and lifestyle factors such as alcohol and smoking status are obtained. All current medications including over-the-counter and herbal medication are documented. Current HbA_1c_ and renal function are checked with venepuncture and local laboratory analysis.

#### Study 2 baseline examination

Examinations are performed in the morning after a 10 h overnight fast. Participants remain on their usual non-diabetes medications; metformin, if used, is stopped for the 24 h preceding the study visit and restarted immediately after. As with Study 1 (described above), anthropometric, diet and quality of life data, blood pressure, stool samples, toenail clippings and urine samples are collected. An intravenous cannula is inserted into a forearm vein according to local protocols. Baseline blood samples are immediately collected for analysis of GAD and islet antigen-2 antibodies, glucagon-like peptide-1, glucagon, insulin, C-peptide, metabolomics, proteomics, HbA_1c_, DNA and RNA. The participant is also fitted with an accelerometer for measurement of physical activity, sedentary behaviour and sleep over 10 days.

##### MMTT

In addition, fasting samples (MMTT time point 0 min) for glucose, insulin and C-peptide analysis are collected. As part of the MMTT, participants consume 250 ml Fortisip liquid drink (18.4 g carbohydrate per 100 ml) over a period of 2–5 min. Blood samples are collected every 30 min for 2 h for subsequent glucose, insulin and C-peptide assays. A postprandial urine sample is also collected, analysed with a simple dipstick and stored for later C-peptide analysis. As part of the baseline visit, all participants also have an abdominal MRI scan, as described above.

#### Study 2 measurements between visits

Each participant’s diabetes management is continued as normal. For monitoring of beta cell function, participants collect postprandial urine samples every 3 months, which are returned to the study centre for analysis of C-peptide.

#### Study 2 follow-up examinations

Two follow-up study visits are carried out in Study 2, one at 9 months and another at 18 months after the baseline visit. The 9-month follow-up involves a repeated medical assessment, medication review and anthropometric assessment, as documented above for the baseline visit. Fasting blood samples are collected in a manner identical with the baseline visit (excluding a blood sample for RNA processing and analysis). The 18-month follow-up examination is identical with the baseline examination.

## Summary

The IMI DIRECT Study is one of the largest and most comprehensive projects ever undertaken for the discovery and validation of biomarkers for glycaemic deterioration and type 2 diabetes. WP2 of DIRECT focuses on deriving new data and biomaterials from two new prospective cohort studies, as well as assimilating data and materials from existing cohort studies led by members of the DIRECT Consortium. The new studies are designed to: (1) identify persons who are clinically defined as being at high risk of rapid glycaemic deterioration and, by consequence, type 2 diabetes, in whom additional novel biomarkers that inform the prediction of these outcomes can be discovered (Study 1); (2) identify persons with recently diagnosed type 2 diabetes, in whom novel biomarkers for diabetes progression (glycaemic deterioration and failure of therapy) might be found. These studies involve in-depth phenotyping using a range of cutting-edge technologies such as: MRI scans of the liver, pancreas and abdomen; metagenomics of faecal DNA; genomic, epigenomic, transcriptomic, proteomic and metabolomic assessments of blood and urine; estimates of pancreatic insulin secretion and insulin action; and detailed lifestyle assessments. The hypotheses generated using these materials will be tested in specially designed clinical trials, which take place in the second part of the DIRECT Study.

## Electronic supplementary material

Below is the link to the electronic supplementary material.

ESM DIRECT WP2 Contributors (PDF 13 kb)

## Abbreviations

DIRECT: Diabetes Research on Patient Stratification
EU: European Union
fsOGTT: Frequently sampled OGTT
IMI: Innovative Medicines Initiative
MMTT: Mixed-meal tolerance test
WP2: Glycaemic deterioration work package
