# Supplementary material for: Discovery of biomarkers for glycaemic deterioration before and after the onset of type 2 diabetes: rationale and design of the epidemiological studies within the IMI DIRECT Consortium
Source: Diabetologia. 2014 Apr 4;57(6):1132–42. doi: 10.1007/s00125-014-3216-x (PMC4018481; doi:10.1007/s00125-014-3216-x)
Supplement: Supplementary file 1 — (PDF 13 kb) [file 125_2014_3216_MOESM1_ESM.pdf]

Discovery of biomarkers for glycaemic deterioration before and after the onset of type 2 diabetes: ***rationale and design of the epidemiological studies within the IMI DIRECT consortium***

Supplementary Material: DIRECT WP2 Contributors

We thank the following for their contributions to the planning, implementation or conduct of the DIRECT Study:

Kristine Højgaard Allin, The Novo Nordisk Foundation Center for Basic Metabolic Research, Section of Metabolic Genetics, Faculty of Health and Medical Sciences, University of Copenhagen; Leila Antikainen, Department of Medicine, University of Eastern Finland, Finland; Margit Bergström, Department of Endocrinology, Skåne University Hospital Lund, Lund Sweden; Laura Botman, VUmc Diabetes Research Center, Hoorn, the Netherlands; Carolien Boukens, VUmc Diabetes Research Center, Hoorn, the Netherlands; Louise Cabrelli, Clinical Research Centre, Ninewells Hospital, Dundee, UK; Ian Clarke, Blood Sciences, Royal Devon and Exeter NHS Foundation Trust, Exeter, UK; Ferrina Clerc, VUmc Diabetes Research Center, Hoorn, the Netherlands; Rebecca Crofts, Blood Sciences, Royal Devon and Exeter NHS Foundation Trust, Exeter, UK; Adrian Cudmore, Blood Sciences, Royal Devon and Exeter NHS Foundation Trust, Exeter, UK; Anders Dahlin, Department of Clinical Sciences, Internal Medicine Research Unit, Lund University, Malmö, Sweden; Jill Ducker, Diabetes Research Network, Clinical Research Facility, Royal Victoria Infirmary, Newcastle-upon-Tyne, UK; Kaija Eirola, Department of Medicine, University of Eastern Finland, Finland; Jannet Entius, VUmc Diabetes Research Center, Hoorn, the Netherlands; Julie Fitzpatrick, Metabolic and Molecular Imaging Group, MRC Clinical Science Centre, Imperial College Hammersmith Campus, London, UK; Heather Ford, Nutrition and Dietetic Research Group, Devision of Diabetes, Endocrinology and Metabolism, Imperial College London, London, UK; Annemette Forman, The Novo Nordisk Foundation Center for Basic Metabolic Research, Section of Metabolic Genetics, Faculty of Health and Medical Sciences, University of Copenhagen; Leif Groop, Department of Clinical Sciences, Diabetes and Endocrinology, Lund University and Lund University Diabetes Centre, Malmö, Sweden; Margit Heier, Department of Epidemiology II, Helmholtz Zentrum München, Neuherberg, Germany; Tarja Heikkinen , Department of Medicine, University of Eastern Finland, Finland; Michelle Hudson, NIHR Exeter CRF, University of Exeter Medical School, Exeter, UK; Aija Jantunen, Department of Medicine, University of Eastern Finland, Finland; Piotr Jaroslaw Chmura, Center for Biological Sequence Analysis, Technical University of Denmark, Denmark; Diane Jarvis, NIHR Exeter CRF, University of Exeter Medical School, Exeter, UK; Bosman Jolanda, VUmc Diabetes Research Center, Hoorn, the Netherlands; Debbie Keetch, NIHR Exeter CRF, University of Exeter Medical School, Exeter, UK; Marney Keiller, Clinical Research Centre, Ninewells Hospital, Dundee, UK; Maria Klintenberg, Department of Endocrinology, Skåne University Hospital Malmö, Malmö Sweden; Jasmina Kravic, Department of Clinical Sciences, Diabetes and Endocrinology, Lund University and Lund University Diabetes Centre,

Malmö, Sweden; Teemu Kuulasmaa, Department of Medicine, University of Eastern Finland, Finland; Seija Laitinen, Department of Medicine, University of Eastern Finland, Finland; Joanne Lawson, Diabetes Research Network, Clinical Research Facility, Royal Victoria Infirmary, Newcastle-upon-Tyne, UK; Heather Loftus, Clinical Research Centre, Ninewells Hospital, Dundee, UK; Tina Hvidtfeldt Loretnzen, The Novo Nordisk Foundation Center for Basic Metabolic Research, Section of Metabolic Genetics, Faculty of Health and Medical Sciences, University of Copenhagen; Tonia Ludwig, Department of Epidemiology II, Helmholtz Zentrum München, Neuherberg, Germany; David Marshall, Blood Sciences, Royal Devon and Exeter NHs Foundation Trust, Exeter, UK; Christa Meisinger, Department of Epidemiology II, Helmholtz Zentrum München, Neuherberg, Germany; Karen Melham, HeLEX, Nuffield Department of Population Health, University of Oxford, UK; Celia Miller, Clinical Research Facility, Royal Victoria Infirmary, Newcastle-upon-Tyne, UK; Marian Nannings, VUmc Diabetes Research Center, Hoorn, the Netherlands; Peter Nilsson, Department of Clinical Sciences, Internal Medicine Research Unit, Lund University, Malmö, Sweden; Sari Olkkonen, Department of Medicine, University of Eastern Finland, Finland; Annette Peters, Department of Epidemiology II, Helmholtz Zentrum München, Neuherberg, Germany; Anna Pålsson, Department of Endocrinology, Skåne University Hospital Lund, Lund, Sweden; Agatha Reus, VUmc Diabetes Research Center, Hoorn, the Netherlands; Rachel Rider, Diabetes Research Network, Clinical Research Facility, Royal Victoria Infirmary, Newcastle-upon-Tyne, UK; Kim Rowden, NIHR Exeter CRF, University of Exeter Medical School, Exeter, UK; Sonja Ruhnke, Department of Clinical Sciences, Genetic and Molecular Epidemiology Unit, Skåne University Hospital Malmö, Lund University, Malmö, Sweden; Sylvia Ruiter, VUmc Diabetes Research Center, Hoorn, the Netherlands; Eila Ruotsalainen, Department of Medicine, University of Eastern Finland, Finland; Ulla Ruotsalainen, Department of Medicine, University of Eastern Finland, Finland; Emil Rydza, Center for Biological Sequence Analysis, Technical University of Denmark, Denmark; Päivi Rytkönen, Department of Medicine, University of Eastern Finland, Finland; Ann-Marie Räihä, Department of Endocrinology, Skåne University Hospital Lund, Lund, Sweden; Raija Räisänen, Department of Medicine, University of Eastern Finland, Finland; Kevin Short, Diabetes Research Network, Clinical Research Facility, Royal Victoria Infirmary, Newcastle-upon-Tyne, UK; Tiina Sistonen, Department of Medicine, University of Eastern Finland, Finland; Stephen Spaull, NIHR Exeter CRF, University of Exeter Medical School, Exeter, UK; Hans Henrik Stærfeldt, Center for Biological Sequence Analysis, Technical University of Denmark, Denmark; Patrycja Stefaniuk, Blood Sciences, Royal Devon and Exeter NHS Foundation Trust, Exeter, UK; Lindsey Taylor, Blood Sciences, Royal Devon and Exeter NHS Foundation Trust, Exeter, UK; E. Louise Thomas, Metabolic and Molecular Imaging Group, MRC Clinical Science Centre, Imperial College Hammersmith Campus, London, UK; Claire Thorne, NIHR Exeter CRF, University of Exeter Medical School, Exeter, UK; Gabi Tjeerdsma, VUmc Diabetes Research Center, Hoorn, the Netherlands; Anne Toivanen, Department of Medicine, University of Eastern Finland, Finland; Andrea Tura, National Research Council, Institute of Biomedical Engineering, Italy; Pauli Vainio, Department of Radiology, Kuopio University Hospital, Finland; Dianne Wake, Diabetes Research Network, Clinical Research

Facility, Royal Victoria Infirmary, Newcastle-upon-Tyne, UK; Josette van Driel, VUmc Diabetes Research Center, Hoorn, the Netherlands; Ritva Vanninen, Department of Radiology, Kuopio University Hospital, Finland; Marianne Veeken, VUmc Diabetes Research Center, Hoorn, the Netherlands; Linda Weijers, VUmc Diabetes Research Center, Hoorn, the Netherlands; Ingrid Wetterholtz, Department of Endocrinology, Skåne University Hospital Malmö, Malmö, Sweden; John Vidler, Blood Sciences, Royal Devon and Exeter NHS Foundation Trust, Exeter, UK; Ulla Viinikanoja, Department of Medicine, University of Eastern Finland, Finland.
